# Selective phenol methylation to 2,6-dimethylphenol in a fluidized bed of iron-chromium mixed oxide catalyst with o–cresol circulation

**DOI:** 10.1186/s13065-014-0051-6

**Published:** 2014-09-16

**Authors:** Witold Żukowski, Gabriela Berkowicz, Jerzy Baron, Stanisław Kandefer, Dariusz Jamanek, Stefan Szarlik, Zbigniew Wielgosz, Maria Zielecka

**Affiliations:** Cracow University of Technology, Faculty of Chemical Engineering and Technology, ul. Warszawska 24, 31-155 Cracow, Poland; Industrial Chemistry Research Institute, ul. Rydygiera 8, 01-793 Warsaw, Poland

**Keywords:** 2,6-dimethylphenol, Fluidized bed, Iron-chromium catalyst

## Abstract

**Background:**

2,6-dimethylphenol (2,6-DMP) is a product of phenol methylation, especially important for the plastics industry. The process of phenol methylation in the gas phase is strongly exothermic. In order to ensure good temperature equalization in the catalyst bed, the process was carried out using a catalyst in the form of a fluidized bed - in particular, the commercial iron-chromium catalyst TZC-3/1.

**Results:**

Synthesis of 2,6-dimethylphenol from phenol and methanol in fluidized bed of iron-chromium catalyst was carried out and the fluidization of the catalyst was examined. Stable state of fluidized bed of iron-chromium catalyst was achieved. The measured velocities allowed to determine the minimum flow of reactants, ensuring introduction of the catalyst bed in the reactor into the state of fluidization. Due to a high content of o-cresol in products of 2,6-dimethylphenol synthesis, circulation in the technological node was proposed. A series of syntheses with variable amount of o-cresol in the feedstock allowed to determine the parameters of stationary states.

**Conclusion:**

A stable work of technological node with o-cresol circulation is possible in the temperature range of350-380°C, and o-cresol_in_/phenol_in_ molar ratio of more than 0.48. Synthesis of 2,6-DMP over the iron-chromium catalyst is characterized by more than 90% degree of phenol conversion. Moreover, the O-alkylation did not occur (which was confirmed by GC-MS analysis). By applying o-cresol circulation in the 2,6-DMP process, selectivity of more than 85% degree of 2,6-DMP was achieved. The participation levels of by-products: 2,4-DMP and 2,4,6-TMP were low. In the optimal conditions based on the highest yield of 2,6-DMP achieved in the technological node applying o-cresol circulation, there are 2%_mol_. of 2,4-DMP and 6%_mol_. of 2,4,6-TMP in the final mixture, whereas 2,4,6-TMP can be useful as a chain stopper and polymer’s molar mass regulator during the polymerization of 2,6-DMP.

## Background

A wide range of applications of products of phenol methylation makes that the process of their preparation the subject of numerous studies [1-23]. Strong bacteriostatic [24], bacteriocidal [25] and fungicidal properties [26] result in methyl-substituted derivatives of phenol being used as preservatives in the food industry [27], antimicrobial agents in the pharmaceutical industry [28], in decontamination and disinfection agents, such as Lysol, creolin [29] and also in the production pesticides [30].

2,6-dimethylphenol (2,6-DMP) is an important product of phenol methylation, especially for the plastics industry. A condensation of 2,6-DMP molecules takes place in the para position, because of locked *ortho* positions. The oxidative polymerization of this derivative of phenol leads to the formation of polyphenylene oxide (PPO) [31], possessing excellent mechanical, dielectric and chemical properties [32]. Thus the PPO is applied in the automotive, electronics, electrical, building, and medical industries [32-35]. Apart from the production of PPO, 2,6-dimethylphenol is also used in the production of medicaments [36,37], pigments [38] and antioxidants [39].

There are known methods of preparation of 2,6-dimethylphenol, both in the liquid phase [1-3] and in the gaseous phase [4-16]. Carrying out the process in the liquid phase is not technologically preferred because of the long reaction time and the necessity of applying high pressure. An additional difficulty is the necessity to separate the catalyst from the products and unreacted substrates [17]. These drawbacks cause the synthesis of 2,6-DMP to be mostly carried out in the gas phase. The reaction of phenol alkylation with methanol is carried out in the presence of various types of catalysts from the group of oxides, mixed oxides [4-9], spinels [10-13,19,20], and zeolites [14-16].

The process of phenol methylation in the gas phase is strongly exothermic (ΔH^o^_r_ = -134,8 kJ/mol_2,6DMP_) and the adiabatic temperature rise equals 425°C for the process carried out under stoichiometric conditions at 330°C. In order to ensure good temperature equalization in the catalyst bed, it was proposed to carry out the process in a catalyst in the form of a fluidized bed. Intensive mixing in the fluidized bed catalyst allows maximum utilization of the catalyst surface and good temperature control, as well as ensures good heat and mass transfer at low pressure drop [40]. It is known that iron oxide forms are part of catalysts for the phenol alkylation [20-23]. A catalyst TZC-3/1 is one of the industrial iron oxide catalysts. This catalyst is produced by Zakłady Azotowe in Tranów, Poland, and is intended for high-temperature conversion of carbon oxide with water vapor in the processes of obtaining hydrogen, syngas and ammonia [41]. Preliminary research has indicated that the phenol methylation on this catalyst selectively leads to products of C-alkylation.

The purpose of this study is to investigate the influence of process parameters on the synthesis of 2,6-dimethylphenol, and to identify the main and simultaneous reactions, as well as to develop a method for on-line monitoring of the reaction extents and to define the parameters of a stable work of 2,6-DMP node with a o-cresol circulation.

## Experimental phase

### Preparation of the catalyst TZC-3/1 fraction to obtain a stable fluidized-bed

**Figure 1:**
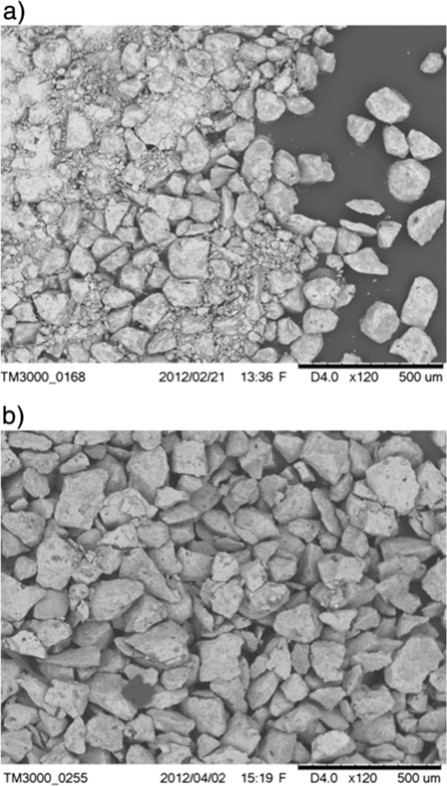
**Powders of the iron-chromium catalyst, a) 75-150 μm fraction obtained after mechanical separation on sieves, b) 75-150 μm fraction after pneumatic removal of the finest fraction.**

**Figure 2:**
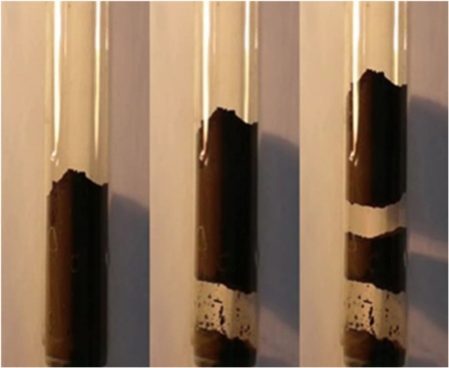
**Slugging of iron-chromium catalyst.**

**Figure 3:**
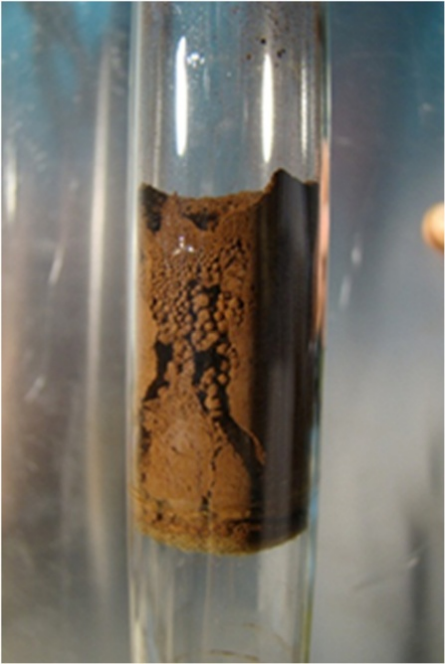
**Channeling of iron-chromium catalyst.**

Achievement of a stable state of a fluidized bed of powdered solids mainly depends on individual characteristics of the powder such as: particle size, density of the material and size of the inter-granular interactions forces [42]. The catalyst used in the studies is obtained in the process of co-precipitation. In order to give it a utility value, the manufacturer of the catalyst produces the powder in the shape of cylindrical pellets (φ = 6 mm, h = 6 mm). The commercial pellets of TZC-3/1 catalyst were subjected to mechanical milling and segregation on sieves. The fraction of catalyst with a grain size of 75-150 μm was isolated (Figure 1a). The mechanical segregation using only the force of gravity was not enough to obtain sufficiently pure fractions of the target particle size. The intermolecular interactions of grains smaller than 75 μm proved to be so large, that it was necessary to purify the fraction 75–150 μm using a specially developed pneumatic method (Figure 1b). The pneumatic purification prevented agglomeration of catalyst particles, such as: slugging (Figure 2), and channeling (Figure 3), that have been observed without additional operation. The composition and density of the catalyst fraction used in further studies are shown in Table 1.

**Table 1:** **Parameters of TZC-3/1 catalyst**

**Bed of TZC-3/1 catalyst, which was used during 2,6-DMP syntheses**	
Mass, g	50
Bulk density, kg/m^3^	1 017 ± 1
Pycnometric density, kg/m^3^	3 602 ± 4
Granular fraction, μm	75 – 150
Fe_2_O_3_, %mas	72 – 90
Cr_2_O_3_, %mas	7.0 – 11.5
CrO_2_, %mas	0 – 0.095
CuO, %mas	1.5 – 4.0

Particle size of 75–150 μm after pneumatic purification.

### Online monitoring of process of 2,6-DMP synthesis on TZC-3/1 catalyst

It can be predicted that the gas mixture leaving the reactor will contain: products of the methylation of aromatics, inorganic and organic components derived from the reaction of catalytic decomposition of methanol as well as unreacted substrates. Due to the ability of these compounds (except H_2_) to absorb electromagnetic radiation in the infrared range, it was possible to carry out quantitative analysis of the reaction mixture’s composition at intervals of several seconds using a FTIR spectrometer.

**Figure 4:**
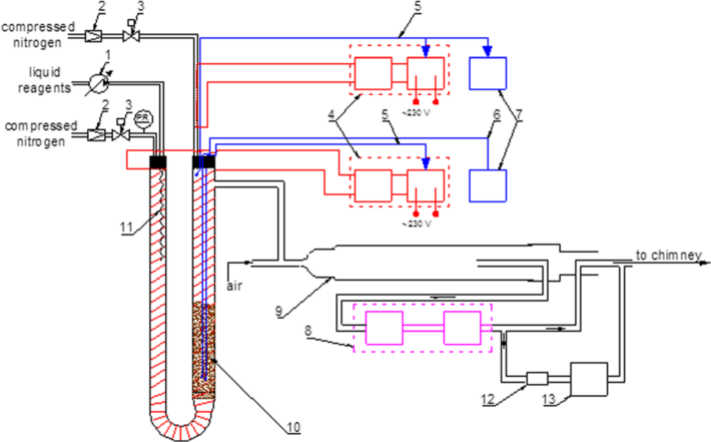
**Scheme of laboratory installation for online monitoring of the synthesis of 2,6-DMP on the iron-chromium catalyst.** 1 – infusion pump, 2 – reducing valve, 3 – control valve, 4 – temperature control system, 5 – regulating thermocouple, 6 – control thermocouple, 7 – recorder/screen, 8 – FTIR, 9 – dilution chamber, 10 – catalyst., 11 – vaporizer, 12 – sampler, 13 – GC/MS analyzer.

The installation diagram is shown in Figure 4. A U-shape reactor served as both a evaporation node of the liquid reactants (left arm) and a chemical reactor with the fluidized bed of catalyst (right arm). The external diameters were 2.3 cm and 3.4 cm, respectively for the left and right arm. In the place of catalyst layer formation, the internal diameter was 3.1 cm. The external wall of the reactor was almost entirely wrapped by a heating spiral. Temperature control was performed using three thermocouples: regulating thermocouple, which was placed 2 cm above the distributor and two measuring thermocouples, which were placed 2.5 cm above the distributor and 1 cm above the gas outlet side.

The Gasmet DX-4000 apparatus (with the firmware) was used for the analysis of the composition of the after-reaction gaseous mixture. It contains a Michelson interferometer and a gas cuvette with an optical path length of 5.0 m, preceded by particulate filter and heated to 180°C. This apparatus allows for obtaining the IR spectrum of the sample in the wave number range of 800-4000 cm^−1^. High temperature of 2,6-DMP synthesis and high product concentration in the exhaust gases resulted in the necessity of diluting post-reaction gases in two-steps with inert gases, by simultaneous cooling down to about 160°C, prior to introduction of the gaseous products into the analyzer. Such diluted mixture containing all the products of the reaction was passed through the demister, reheated to 180°C and directed to the gaseous cuvette of FTIR analyzer after filtration on ceramic and Teflon filters.

### Evaluation of the synthesis by FTIR analysis

**Figure 5:**
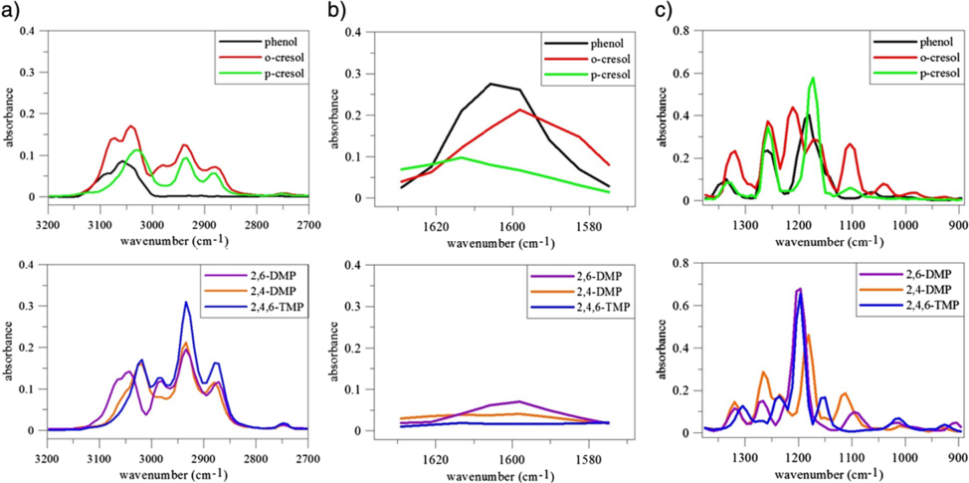
**Spectra of aromatic products of the process in three selected ranges of wave number: a) 3200-2700 cm** ^**-1**^ **, b) 1636-1568 cm** ^**-1**^ **, c) 1380-895 cm** ^**-1**^ **.**

The correct FTIR analysis of the reaction mixture requires a comparison of the reference spectra of all possible process components with the obtained one. The library of spectra, which are a component of the FTIR software, were used for some of the substances (water, CO, CO_2_, C_x_H_y_, HCHO). The spectra for the aromatic compounds were made and calibrated in the concentration range resulting from their expected concentrations in the reaction mixture with an accepted degree of dilution. After removal of the pump, the vaporizer and the catalyst, the installation created for 2,6 synthesis was used for the purpose of obtaining spectra of aromatic compounds and their calibration. A specific amount of substance was placed in the reactor and then pulsed heated in such a way, as not to exceed the absorbance value of 0.8. The procedure was performed six times for each substance. It was assumed that the measured absorbance value corresponds to a concentration of 1000 ppm, which made it possible to show results in the form of a graph of changes in concentration of the substance at the time of evaporation. The areas under the curve were converted to amounts of the substance. The quotient of the calculated quantity of substance to quantity introduced into the reactor gave a correlation coefficient which allows to give the actual value for the assumed value of concentration (1000 ppm). Structural similarity of methyl derivatives of phenol makes their IR spectra similar. Therefore, three ranges of infra-red analysis were selected for the aromatic compounds and they were used at the same time during the analysis. The differences in the spectra of aromatic compounds in the selected analysis ranges are shown in Figure 5. The analysis ranges for all reagents are shown in Table 2. Such choice of analysis conditions allowed correct results to be obtained, which was confirmed by low value of the residual IR spectrum in additional GC/MS analysis. Gas chromatograph analysis was periodically performed using PerkinElmer Clarus 500 coupled with mass detector. The sample was aspirated by two series-connected probes/samplers containing sorbent XAD - 7 [43]. Absorbed substances were extracted with methanol in an ultrasonic chamber. Allocation was performed on a Rtx-35 MS column, measuring 30 m x 0.25 mm x 0.25 microns. Oven Temperature program includes the following steps: 35°C for 2 min; 12°C/min to 180°C; 30°C/min to 280°C, 280°C for 2.58 min. Ions with a ratio m/z corresponding to the molecular ions of phenol (m/z = 94), cresols and anisole (m/z = 108), dimethylphenols (m/z = 122) and trimetylphenols (m/z = 136) were selected. Moreover, scanning was performed for m/z from 50 to 300 m in order to detect other impurities. The retention times of the components are summarized in Table 2. Anisole - the product of phenol O-alkylation was not included as a reference spectrum for the method of FTIR analysis, because preliminary tests with the GC-MS analyzer showed that it does not occur in the reaction products on the TZC-3/1 catalyst.

**Table 2:** **Selected ranges for FTIR analysis and the retention times of GC-MS analysis of components of the process**

**Substance**	**Range of FTIR analysis, cm** ^**−1**^	**Retention time, min**
Water	4200-3800	-
Carbon dioxide	3700-3400	-
Propane	3300-2600	-
Ethane	3100-2800	-
Methane		-
Formaldehyde	2900-2500	-
Carbon monoxide	2200-2000	-
Methanol	1500-900	-
Ethylene	1200-900	-
Anisole	-	6.90
Phenol	see figure 5	7.85
o-cresol	see figure 5	8.88
m-cresol	see figure 5	9.13
p-cresol	see figure 5	9.14
2,6-DMP	see figure 5	9.63
2,5-DMP	see figure 5	10.06
2,4-DMP	see figure 5	10.08
2,3-DMP	see figure 5	10.58
2,4,6-TMP	see figure 5	10.80
2,3,5-TMP	see figure 5	11.64
3,4,5-TMP	see figure 5	12.28

## Experimental

### Determination of the minimum fluidization velocity

It was assumed that evaporated mixture of reagents: phenol, methanol and water, will affect the fluidization agent of the catalyst in the reactor. The reactant flow could not be too large, because its increase causes reduction of contact time between the catalyst and the reactants, but it had to be large enough to ensure stable state of fluidization of the catalyst. Moreover, the volume fraction of bubbles in the catalytic bed increase with rising gas velocity, making it a factor in reducing process efficiency. This results from the fact that the processes in the interiors of bubbles running without contact with the solid phase. This meant that the substrates should be fed to the reactor at a volume flow, providing that their velocity is slightly higher than the minimum fluidization velocity.

**Figure 6:**
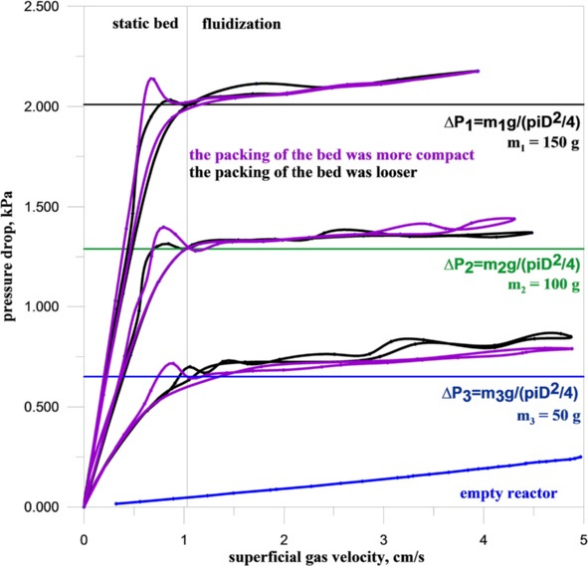
**Pressure drop as a function of superficial gas velocity at 200°C.**

Many empirical correlation patterns determining the minimum fluidization velocity are known [44], however the U_mf_ velocity of the TZC-3/1 catalyst was experimentally determined because of a substantive divergence in the results of the abovementioned correlations obtained for the same gas flow through the bed. The minimum fluidization velocity was determined by placing catalyst beds with different masses: 50, 100 and 150 g, and with varying degrees of packing in a reactor with a 30 mm diameter. The created layer of catalyst had static high, respectively (loose bed/compact bed): 66/64, 132/128 and 195/191 mm. Gas at temperatures from 22°C to 300°C was passed through the catalyst. An example of a relationship between pressure drop and the fluidizing medium velocity at 200°C is shown in Figure 6. Minimum fluidization velocities at other temperatures were determined from an analogous graph, and summarized in Table 3. It was verified that the degree of preliminary packing of the bed did not significantly effect on the value of U_mf_, which means that cohesive forces in this pneumatically purified material are not a barrier in the process of fluidization of such prepared catalyst. The measured velocities allowed to determine the minimum flow of reactants, ensuring the introduction of the catalyst bed in the reactor into the state of fluidization.

**Table 3:** **Minimum fluidization velocities of the catalyst TZC-3/1**

**Bed temperature of catalyst, °C**	**Minimum fluidization velocity, cm/s**
22	1.08
55	0.98
120	0.83
200	0.94
300	0.74

### Synthesis of 2,6-dimethylphenol from a mixture of phenol:methanol:water in molar ratio 1:5:1

**Figure 7:**
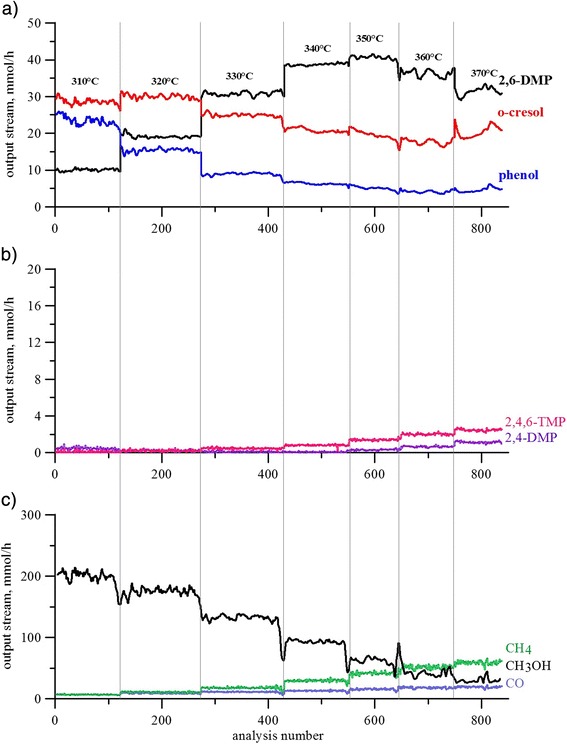
**Output molar stream during the synthesis of 2,6-DMP from mixture phenol:methanol:water as 1:5:1. a)** phenol, o-cresol, 2,6-DMP **b)** 2,4-DMP, 2,4,6-TMP **c)** methanol, CO, CO_2_, CH_4_ **.**

The synthesis process was started when the catalyst layer, externally heated and washed with a stream of nitrogen, achieved 310°C. Then, a solution containing phenol, methanol and water in a molar ratio 1:5:1 was introduced into the evaporator. Presence of water in the mixture of substrates has a beneficial effect in maintaining a high catalyst activity. The solution was fed into the evaporator at a rate of 20 cm^3^/h, ensuring the achievement of a stable fluidized state at the lowest temperature of the catalyst layer. That meant, that 64.1 mmol/h of phenol, 320.5 mmol/h of methanol and 64.1 mmol/h of water were fed to the reactor. The analysis of the composition of the post-reaction gases was performed every 7 seconds using a FTIR spectrometer. The process was carried out in stages, by increasing the temperature of the catalyst layer in 10°C increments, to the point when temperature change did not contribute to the increase in concentration of 2,6-DMP. The selected streams of post-reaction components are shown in Figure 7.

With increased bed temperature, the molar stream of phenol in the post-reaction gases decreased (Figure 7) reaching the value about 6 mmol/h at 360°C, which corresponds to 90% consumption of this substrate. The molar stream of o-cresol - the first product of phenol methylation initially went up and went down because of subsequent alkylation after reaching 320°C. The p-cresol was not found in the product (neither by FTIR nor GC/MS analysis). The maximum yield of 2,6-DMP stream was achieved at 350°C. The streams of 2,4-DMP and 2,4,6-TMP by-products increased, but this increase was small (Figure 7b). At 350°C, the temperature of maximum 2,6-DMP yield, the total stream of 2,4-DMP and 2,4,6-TMP was approximately 24 times smaller than the stream of the target aromatic product.

**Figure 8:**
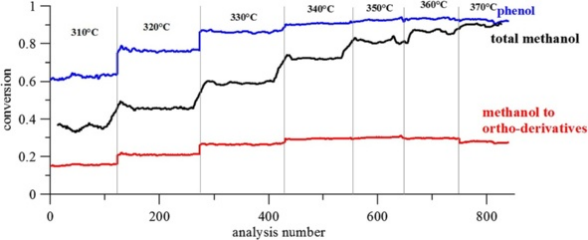
**Degrees of conversion of phenol, methanol and methanol to ortho-substituted products.**

The method of evaluating the course of reactions using infrared analyzer made it possible to follow the parallel process of methyl alcohol degradation. The output stream of methanol (Figure 7c) significantly decreased with temperature rise, while the phenol stream achieved a relatively constant value above 340°C (Figure 7a). The conversions of both substrates are summarized in a single graph (Figure 8) to illustrate the degree of loss of methanol due to the competitive reaction of decomposition. The conversions of phenol (blue line) and methanol (total - black and to ortho-substituted products - green) were shown. It is seen that above 340°C, methanol is mainly consumed in the decomposition and reduced to gaseous components.

Parametric evaluation of process such as phenol conversion, selectivity of ortho-substituted products, and yield of 2,6-DMP, was calculated from the average value of streams of post-reaction gases. The calculation was based on the following formulas, where the inlet and outlet streams of substance were marked by the name of the individual compound.

Conversion of phenol:

$$ {C}_{Ph}=100\;\%\cdot \left(1-\frac{pheno{l}_{out}}{pheno{l}_{in}}\right) $$

Selectivity of 2,6-dimethylphenol:

$$ {\boldsymbol{S}}_{\boldsymbol{2},\boldsymbol{6}\boldsymbol{\hbox{-}}\boldsymbol{D}\boldsymbol{M}\boldsymbol{P}}=\frac{100\%\cdot 2,6-DM{P}_{out}}{2,6-DM{P}_{out}+o- creso{l}_{out}+2,4-DM{P}_{out}+2,4,6-TM{P}_{out}} $$

Selectivity of o–cresol:

$$ {\boldsymbol{S}}_{\boldsymbol{o}\boldsymbol{\hbox{-}}\boldsymbol{c}}=\frac{100\%\cdot o- creso{l}_{out}}{2,6-DM{P}_{out}+o- creso{l}_{out}+2,4-DM{P}_{out}+2,4,6-TM{P}_{out}} $$

Yield of 2,6-dimethylphenol:

$$ {\boldsymbol{Y}}_{\boldsymbol{2},\boldsymbol{6}\boldsymbol{\hbox{-}}\boldsymbol{D}\boldsymbol{M}\boldsymbol{P}}=\frac{100\%\cdot 2,6-DM{P}_{out}}{pheno{l}_{in}+o- creso{l}_{in}} $$

Results of the synthesis, calculated according to above formulas, are shown in Table 4. The highest 63% yield of 2,6-DMP was achieved at 350°C, with over 92% conversion of phenol. The reduced yield of 2,6-DMP in the presence of high degree of phenol conversion was caused by high content of o-cresol in products of the process. 1/3 of aromatic products consisted of o-cresol. The temperature rise did not lead to an increase in 2,6-DMP efficiency due to unavoidable methanol decomposition causing deficiency of the alkylating agent. Increase in the amount of methanol in the feedstock is one of the possible remedial measures.

**Table 4:** **Results of 2,6-DMP synthesis from mixture phenol:methanol:water in molar ratio 1:5:1**

**Temperature of bed**	**Conversion of phenol [%]**	**Selectivity of 2,6-DMP [%]**	**Selectivity of o-krezol [%]**	**Yield of 2,6-DMP [%]**
310°C	63.50	25.56	73.05	15.62
320°C	76.06	38.57	60.49	29.84
330°C	86.19	54.72	44.23	48.03
340°C	90.18	64.08	34.42	60.39
350°C	92.17	65.58	31.63	62.90
360°C	93.63	64.08	31.19	56.62
370°C	92.83	56.69	36.79	49.05

Feed flows: 64.1 mmol/h of phenol, 320.5 mmol/h of methanol and 64.1 mmol/h of water, Pressure: 1 atm.

### Synthesis of 2,6-dimethylphenol from a mixture of phenol:methanol:water in molar ratio 1:8:1

**Figure 9:**
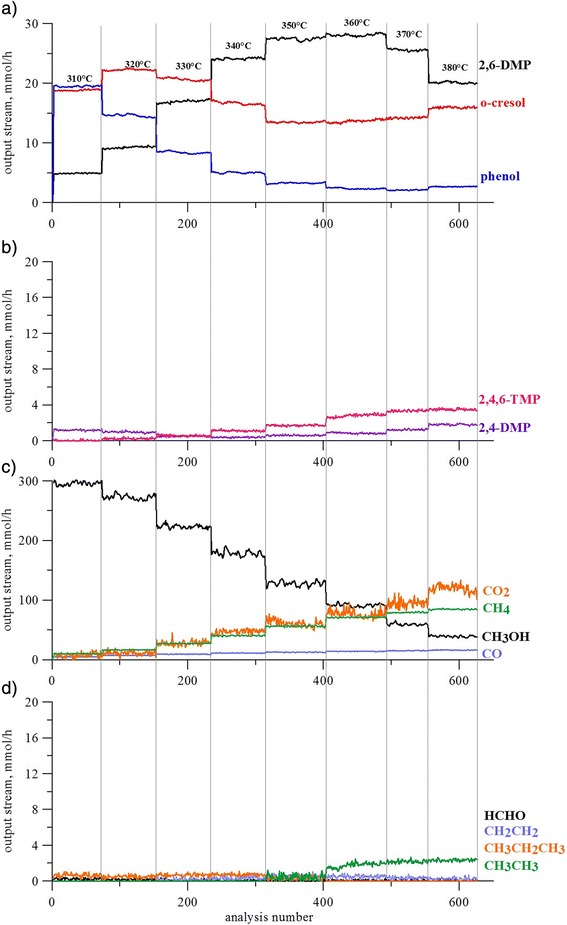
**Output mass fluxes during the synthesis of 2,6-DMP from mixture phenol:methanol:water as 1:8:1. a)** phenol, o-cresol, 2,6-DMP **b)** 2,4-DMP, 2,4,6-TMP **c)** methanol, CO, CO_2_, CH_4_ **d)** methane, ethane, ethylene, formaldehyde.

Solution of phenol, methanol and water in a molar ratio 1:8:1 was dispensed into the vaporizer at a flow rate of 20 ml/h. This meant that the feed to the reactor contained: 46.5 mmol/h of phenol, 370.7 mmol/h of methanol and 46.4 mmol/h of water. It was found that the maximum participation of 2,6-DMP in products was achieved at 360°C (Figure 9a). Carrying out the process at higher temperature lead to a slight increase in streams of byproducts: o-cresol, 2,4-DMP, 2,4,6-TMP, methane, ethane and carbon dioxide (Figure 9a, b, c). The data in Table 5, which summarize results of the synthesis, shows that not only did the increase in the amount of methanol in the feed not produce the expected increase in the yield of 2,6-DMP, but it decreased selectivity and yield of 2,6-DMP.

**Table 5:** **Results of 2,6-DMP synthesis from mixture phenol:methanol:water in molar ratio 1:8:1**

**Temperatur of bed**	**Conversion of phenol [%]**	**Selectivity of 2,6-DMP [%]**	**Selectivity of o-krezol [%]**	**Yield of 2,6-DMP [%]**
310°C	57.94	19.67	75.42	10.60
320°C	68.52	28.27	68.03	19.96
330°C	81.83	43.81	53.43	36.59
340°C	89.14	57.04	39.39	52.04
350°C	92.94	63.48	31.16	59.23
360°C	94.89	61.86	30.07	60.62
370°C	95.43	57.68	32.02	55.23
380°C	94.21	48.63	38.53	43.39

Feed flows: 46.5 mmol/h of phenol, 370.7 mmol/h of methanol and 46.4 mmol/h of water, Pressure: 1 atm.

Comparison of the conversion and selectivity of 2,6-DMP, obtained at different excess methanol in the reaction mixture with phenol to methanol ratio of 1:5 and 1:8 (Tables 4 and 5) indicates that using a higher excess of methanol as methylating agent in the reaction of phenol with methanol does not increase the degree of conversion of phenol and does not improve the selectivity of 2,6-DMP. It was observed that, the use of a larger excess of methanol in the temperature range of 310-330°C leads to a slightly decreased 2,6-DMP yield and selectivity, and in the range above 330°C the conversion of methanol and selectivity of 2,6-DMP is practically the same at both of the methanol excesses applied. In both cases, the methanol is used in excess with respect to the stoichiometric requirements in the synthesis of 2,6-DMP (in the first case, the excess equals 2.5 and the in the second synthesis equals 4). Increase in excess of methanol significantly reduces the concentration of phenol in the feed, thereby leading to reduction in the rate of reaction, which results in lower conversion of phenol characteristic for the subsequent reactions and in a lower selectivity of dimethyl derivatives in favor of the intermediate product - o-cresol.

In the case of the process, in which, as a result of subsequent reactions in the mixture leaving the reactor, there is a large amount of an intermediate product, it may be more cost-effective to recycle it to the process. In a test arrangement without separating and recycling o-cresol, that kind of state can be created artificially by placing it in the mixture of substrates and selecting conditions for the synthesis process to ensure that the amount of this component before and after passing through the catalyst layer is the same.

The assumption that technology of 2,6-dimethylphenol production using fluidized bed technique should include recovery and o-cresol recycle module, in further considerations results in two areas of analysis: a separate area for the reactor and another for the technological node with o-cresol circulation. The earlier definitions will refer to process step containing only the reactor, where o-cresol is both the substrate and the product. Analysis of the technological node with o-cresol circulation, where o-cresol is treated as neither substrate nor product, will rely on modified definitions of selectivity and yield:

Selectivity of 2,6-dimethylphenol in the technological node of synthesis with o-cresol circulation:

$$ {\boldsymbol{S}}_{\boldsymbol{2},\boldsymbol{6}\boldsymbol{\hbox{-}}\boldsymbol{D}\boldsymbol{M}\boldsymbol{P},\boldsymbol{n}}=\frac{100\%\cdot 2,6-DM{P}_{out}}{2,6-DM{P}_{out}+o- creso{l}_{out}-o- creso{l}_{in}+2,4-DM{P}_{out}+2,4,6-TM{P}_{out}} $$

Yield of 2,6-dimethylphenol in the technological synthesis node with circulation of o–cresol in the system:

$$ {\boldsymbol{Y}}_{\boldsymbol{2},\boldsymbol{6}\boldsymbol{\hbox{-}}\boldsymbol{D}\boldsymbol{M}\boldsymbol{P},\boldsymbol{n}}=\frac{100\%\cdot 2,6-DM{P}_{out}}{pheno{l}_{in}} $$

To determine the state of technological node, factor was introduced and defined as:

$$ \Phi =\frac{o- creso{l}_{in}}{o- creso{l}_{out}} $$

The coefficient value of Φ = 1 means achievement of stationary state by the technological node. The value Φ < 1 means an increase of the o-cresol amount in the technological node, while Φ > 1 means decrease in the amount of o-cresol. When Φ ≠ 1, the value of selectivity and yield in the synthesis node do not have significant sense, because they are calculated at a specific time of unsteady state.

### Synthesis of 2,6-DMP from a mixture of phenol:o-cresol:methanol:water in molar ratio 1:x:8:1

**Figure 10:**
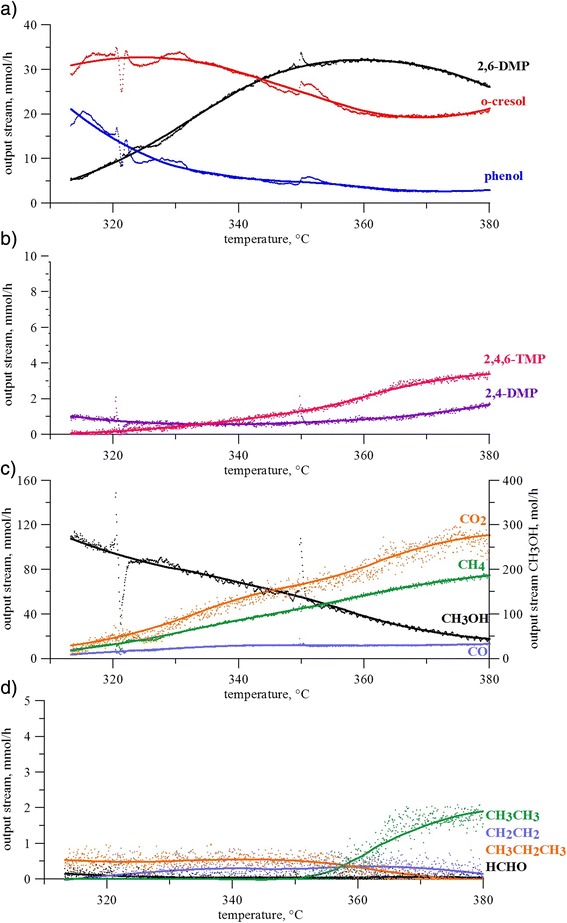
**Output mass fluxes during the synthesis of 2,6-DMP from mixture phenol:o-cresol:methanol:water in molar ratio 1:0.4:8:1. a)** phenol, o-cresol, 2,6-DMP **b)** 2,4-DMP, 2,4,6-TMP **c)** methanol, CO, CO_2_, CH_4_ **d)** methane, ethane, ethylene, formaldehyde.

A series of 2,6-DMP syntheses from a mixture of phenol:o-cresol:methanol:water in a molar ratio 1:x:8:1 was conducted. Previous trials with 1:x:5:1 ratio of reactants did not result in the achievement of a steady state of the technological node with o-cresol circulation in the system. In contrast to the previous attempts, the temperature changes were performed in a continuous manner at a rate of 1°C/min. An example of the observed changes in the output streams for synthesis from a mixture of phenol, o-cresol, methanol and water as 1:0.4:8:1 is shown in Figure 10. Above 360°C, the 2,6-DMP stream decreased with the rise in temperature. This situation is related to increase in the following streams in post-reaction gases: o-cresol, 2,4-DMP and 2,4,6-TMP. Steady state of the node for this feedstock composition has not been obtained, as is clear from the value of coefficient Φ ≠ 1. However, stationary states of technological node with o-cresol circulation (Φ = 1) have been achieved in experiments with the feedstock including 50% and more of o-cresol with respect to phenol.

**Figure 11:**
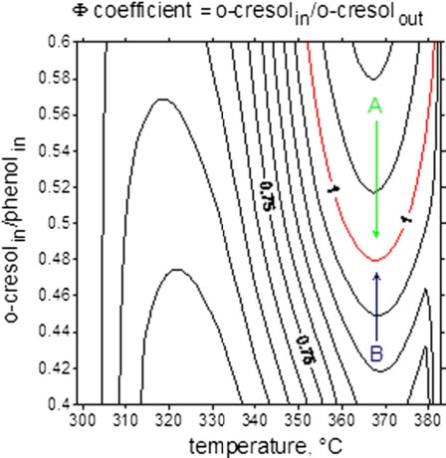
**Dependence of** Φ **coefficient on the temperature and o-cresol**_**in**_ **/phenol**_**in**_ **ratio.**

**Figure 12:**
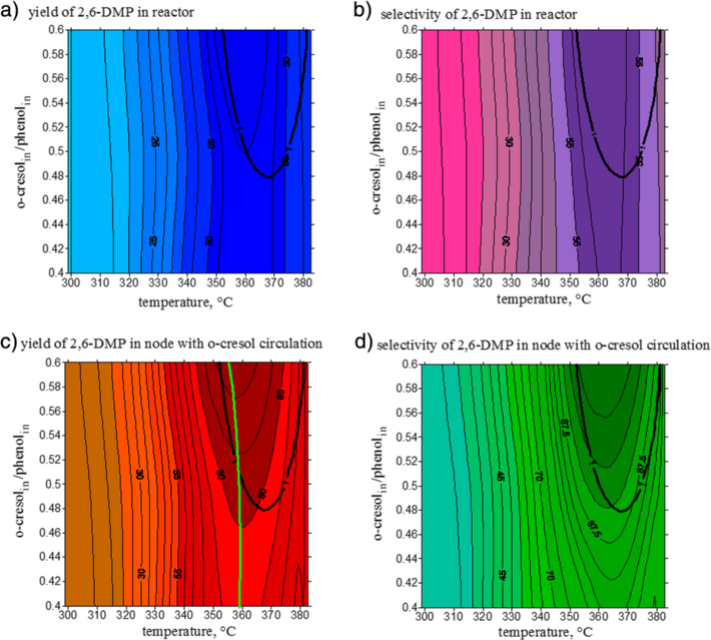
**2,6-DMP efficiency and selectivity in the reactor (a, b) and in the technological node (c, d) during the synthesis from mixture of phenol:o-cresol:methanol:water as 1:x:8:1 as a function of the temperature and the o-cresol**_**in**_ **/phenol**_**in**_ **ratio.** Highlighted lines: the isoline Φ = 1 in black, the maximum productivity of 2,6-DMP in the node in green.

Using a two-dimensional approximation, the results of experiments with variable amounts of o-cresol are shown in form of a graph (Figure 11), which illustrates dependence of coefficient Φ on bed temperature and the o-cresol_in_/phenol_in_ ratio. Isoline Φ = 1, which characterizes steady-state of the node, is highlighted in red. The calculated values of ortho-substituted products selectivity and the yield of 2,6-DMP, both in the reactor and in the technological node, were also presented in the form of analogous graphs in Figure 12a-d. At Φ = 1, the yield and selectivity of 2,6-DMP gain practical significance. For example, in the case of synthesis from a mixture with the phenol:o-cresol ratio of 1:0.5, two points with constant amount of o-cresol set at 360°C and 375°C have been received. The point at 360°C is preferable not only because of the higher yield of 2,6-DMP (82%, at 92% conversion of phenol) (Figure 12c), but also due to the lower temperature and associated with it lower streams of the aromatic by-products.

The green line highlighted in Figure 12c indicats the maximum yield of desired 2,6-DMP. This line crosses the black isoline Φ = 1 at the point of maximum yield of technological node in a stationary state.

In all the experiments, the catalyst loading was equal to 0,353 ± 0,013 g_feed_/(g_catalyst_⋅h). The volumetric flow rate, calculated at 310°C was equal 0,0199 ± 0,0020 m^3^/h. These fluctuations were caused by varying amounts of o-cresol in the feed at the constant liquid flow rate of starting material. The LHSV (liquid hourly space velocity) for all syntheses equals 0.339. During all the presented syntheses of 2,6-DMP, stable fluidization and constant value of pressure drop were observed.

## Discussion

The results of the experiments were presented in a graphic form of operational maps. The maps show dependence of efficiency, conversion, selectivity of selected reagents and the coefficient/factor Φ on bed temperature and o-cresol to phenol molar ratio in the mixture fed to the reactor. The steady-state conditions of the technological node of 2,6-DMP synthesis, stemming from the constant amount of o-cresol circulating in the system were indicated by isoline Φ = 1 in Figure 11. Stable work of technological node is possible in the temperature range 350-380°C, and o-cresol_in_/phenol_in_ molar ratio of more than 0.48. If the system is initially in a non-stationary state of area A, where Φ > 1, the amount of o-cresol in the reactor will be decreased during the process until the system reaches equilibrium. Similarly, if the system is in area B (Φ < 1), then, as time goes by, the amount of o-cresol in the outlet will continuously increase until Φ = 1, which guarantees stability of the node.

**Figure 13:**
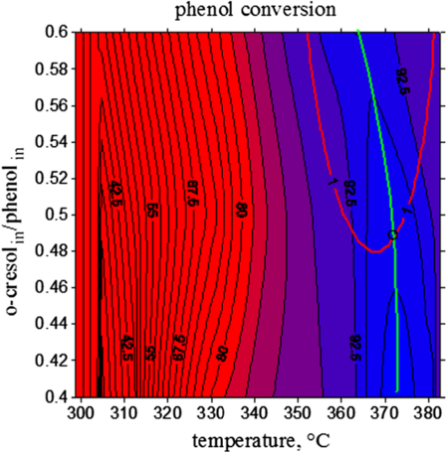
**Phenol conversion as a function of the bed temperature and o-cresol**_**in**_ **/phenol**_**in**_ **ratio during experiments with varying amounts of o-cresol in the feed.** Highlighted lines: the isoline Φ = 1 in red, the maximum phenol conversion in green.

**Figure 14:**
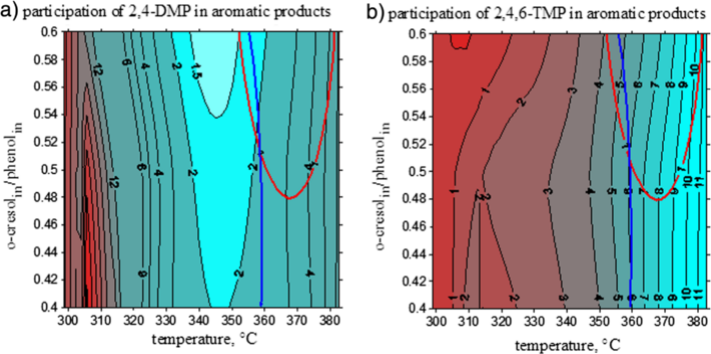
**Percentage of byproducts: 2,4-DMP (a) and 2,4,6-TMP (b) as a function of cresol**_**in**_ **/phenol**_**in**_ **ratio and the bed temperature.** Previously obtained isoline Φ = 1 (red) and maximum conversion of phenol (blue) was applied on maps.

The parameters of stable-work of the node, ensuring maximum yield of 2,6-DMP were also found (Figure 12c). Of course, in this case the conditions of o-cresol_in_/phenol_in_ > 0.48 and bed temperature in the range of 350-380°C were met. It should be stressed, that the application of o-cresol circulation in the synthesis dramatically improved selectivity of the process (compare Figure 12b and d). Instead of maximum yield of 2,6-DMP, the best consumption of phenol may be the basic factor in optimization (Figure 13). The phenol conversion is more than 90% at each point of the isoline of node steady states. The intersection of curves illustrating the steady state of the node (Φ = 1) and maximum usage of phenol can determine the parameters of optimal work of the node (o-cresol_in_/phenol_in_ = 0.49, temperature of catalyst 372°C). On the other hand, the process of 2,6-DMP synthesis carried out in optimal conditions should involve the smallest possible amount of by-products, especially those which cannot be used in further stages of the process. Thus, 2,4-DMP and 2,4,6-TMP participation in the product stream should be taken into consideration during optimization of the 2,6-DMP process (Figure 14a-b). In the optimal conditions based on the highest yield of 2,6-DMP in the node, there are 2%_mol._ of 2,4-DMP and 6%_mol._ of 2,4,6-TMP, whereas 2,4,6-TMP can be useful as chain stopper during polymerization of 2,6-DMP [45,46]. Irrespective of the direction of optimization, the final, optimal point of the reactor operation will always be located on the designated operating isoline Φ = 1.

## Conclusion

Purified fraction of the iron-chromium catalyst with a grain size of 75–150 μm is a material, which undergoes stable fluidization. Experimentally determined minimum fluidization velocity allowed to select the necessary molar flow rate of substrates. Thanks to proper dilution system of the products stream, the laboratory installation enabled online reaction monitoring with an FTIR spectrometer. Wave numbers for the various process components selected in separate proceedings guarantee credible results of the FTIR analysis, confirmed by GC/MS analysis. The problem of high o-cresol content in the products of the 2,6-dimethylphenol synthesis can be solved by development of a technological node with o-cresol circulating in the system. Presented studies allow for determination of stationary conditions of 2,6-DMP synthesis node with o-cresol circulation. Optimal work of the technological node is possible in the temperature range of 350-380°C and with the o-cresol_in_/phenol_in_ molar ratio of more than 0.48. Development of 2,6-DMP technology with o-cresol circulation, depending on temperature, should allow to obtain even 90% yield of 2,6-DMP with more than 90% phenol conversion in stationary conditions defined by plotted on both graphs isoline Φ = 1, which is evident by comparing Figures 12 and 13.
